# IL-4 and IL-4 Receptor Expression Is Dispensable for the Development and Function of Natural Killer T Cells

**DOI:** 10.1371/journal.pone.0071872

**Published:** 2013-08-26

**Authors:** Archna Sharma, Rosa Berga-Bolanos, Dil Afroz Sultana, Jyoti Misra Sen

**Affiliations:** Immune Cells and Inflammation Section, National Institute on Aging, National Institutes of Health, Baltimore, Maryland, United States of America; St. Jude Children's Research Hospital, United States of America

## Abstract

CD4 T cells acquire functional properties including cytokine production upon antigenic stimulation through the T cell receptor (TCR) and differentiate into T helper (Th) cells. Th1 cells produce interferon (IFN)-γ and Th2 cells produce interleukin (IL)-4. Th1 and 2 cells utilize IFN-γ and IL-4 for further maturation and maintenance, respectively. Promyelocytic leukemia zinc finger (PLZF)-expressing invariant natural killer T (iNKT) cells develop in the thymus and acquire functional ability to produce IL-4 and IFN-γ in the thymus in the absence of antigenic stimulation. In response to antigenic stimulation, iNKT cells rapidly produce IFN-γ and IL-4. However, it is still unknown as to whether iNKT cells require these cytokines for maturation or survival in vivo. In this study, using IL-4- and IL-4 receptor- (IL-4R) deficient mice, we demonstrate that IL-4 as well as IL-4R expression is dispensable for the development, function and maintenance of iNKT cells.

## Introduction

The mammalian thymus supports the development of conventional T cells from bone marrow derived precursors. T cells express T cell receptors (TCR) made up of rearranged α and β chains. In addition, the thymus facilitates the development of invariant natural killer T (iNKT) cells that express a limited repertoire of TCR-αβ, characterized by expression of Vα14Jα18 together with Vβ2, 7 or 8.2 in mice, as well as cell surface markers shared with NK cells [1]–[4]. Transcription factor promyelocytic leukemia zinc finger (PLZF), encoded by the *Zbtb16* gene, was recently shown to regulate iNKT cell maturation [5]–[9]. In particular, PLZF confers the capacity to acquire functional capabilities in T cells in the absence of overt antigenic stimulation [7]. Recent studies have shown that iNKT cells pass through an immature developmental stage where they produce IL-4 in apparent absence of stimulation and STAT6 signaling [10]. These studies therefore suggest a role for IL-4 in the development of iNKT cells.

Mature TCR-αβ T cells migrate to the peripheral organs to provide immune protection from invading pathogens as well as tumors. During an immune response, conventional CD4-expressing T cells undergo TCR-induced and cytokine-dependent differentiation into T helper (Th)-1 and Th2 cells [11]–[14]. Th1 cells produce interferon (IFN)-γ and Th2 cells produce interleukin (IL)-4. Importantly, differentiated Th cells utilize the cytokines they produce to promote and maintain their differentiated status [15]–[17]. Innate TCR-αβ iNKT cells, having acquired the ability to rapidly produce both IFN-γ and IL-4 during development in the thymus, rapidly respond to TCR-dependent stimulation by pathogenic antigen [2], [18], [19]. In analogy with Th cells, iNKT cell maintenance might be dependent on autocrine cytokines. However, an earlier study, preceding the usage of CD1d-tetramer to track the iNKT cell population, showed that the IL-4 deficiency did not affect development of HSA^low^CD8^low^CD44^high^NKR-P1^+^ cells [20]. Although it is known that iNKT cells are found in IL-4-deficient mice, it has not been rigorously demonstrated as to whether IL-4 or IL-4R expression on iNKT cells is required for the proper development, function or maintenance of iNKT cells *in vivo*.

In this report, we show that IL-4/IL-4Rα expression is entirely dispensable for the development, function and maintenance of iNKT cells in the thymus and peripheral lymphoid tissues. More specifically, we demonstrate that iNKT cells generated in control and IL-4- or IL-4Rα-deficient mice are comparable in numbers and phenotype in the thymus, spleen, lymph nodes and liver. Finally, we show that iNKT cells generated in IL-4- and IL-4R-deficient mice are functional as they rapidly produce comparable amounts of IFN-γ and IL-4 in vitro as well as in vivo. Thus, data provided in this report demonstrate that IL-4/IL-4R signaling is dispensable for the generation, maintenance of and cytokine production by iNKT cells.

## Results

### IL-4 expression is not required for the development of iNKT cells in the thymus

**Figure 1:**
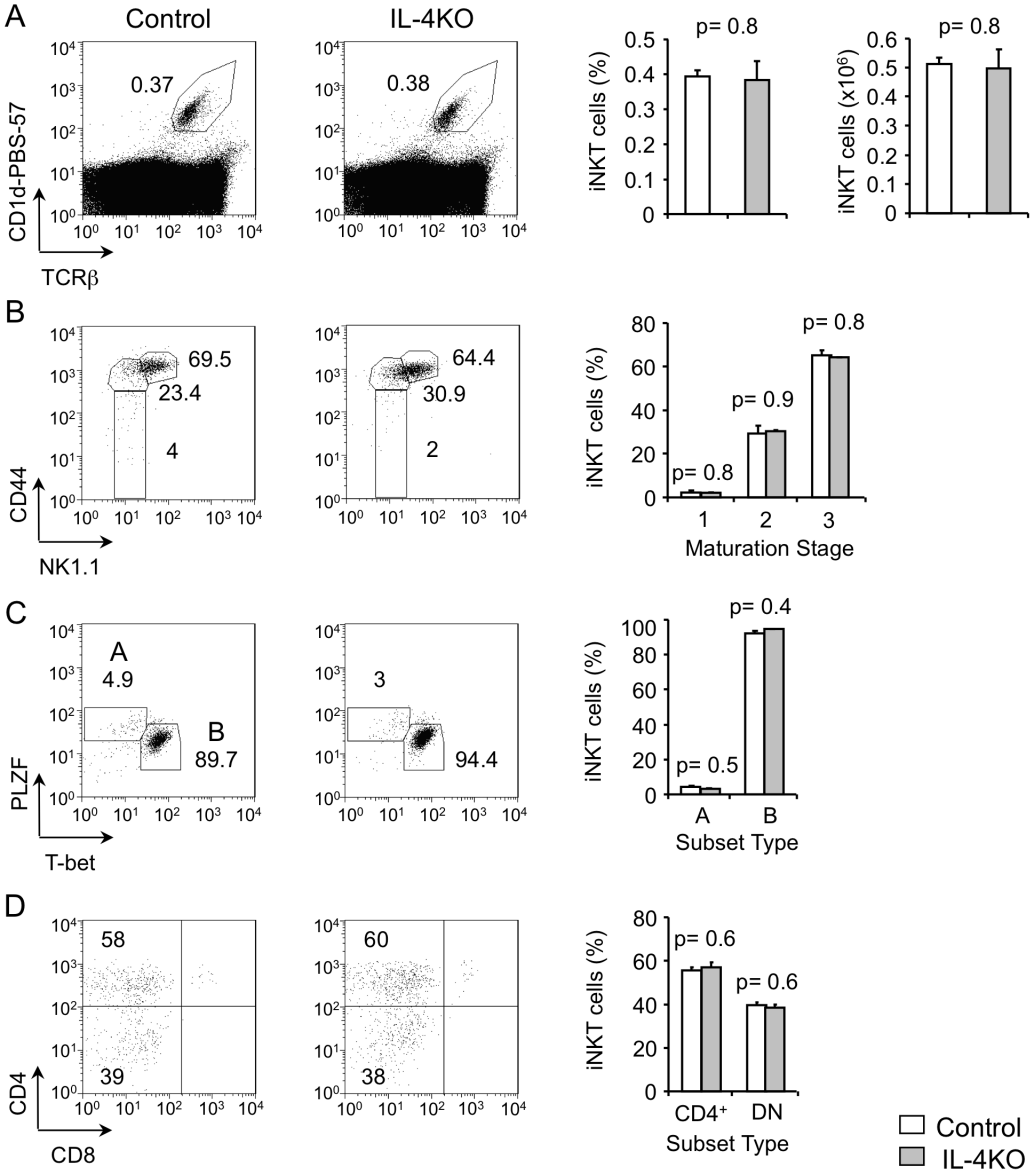
IL-4 is not required for the development of iNKT cells in thymus. (**A**) Flow-cytometric analysis of expression of CD1d-PBS-57 and TCRβ on total thymocytes from control and IL-4KO mice. Numbers adjacent to outlined areas and the graphs show percent and cell numbers of total iNKT cells as indicated. Data are representative of three independent analyses with total six mice per group. **(B)** Flow-cytometric analysis of expression of CD44 and NK1.1 on gated thymic iNKT cells from control and IL-4KO mice. Numbers adjacent to outlined areas and graphs show percent of gated iNKT cells divided into maturation stages 1 (CD44^lo^NK1.1^−^), 2 (CD44^hi^NK1.1^−^) and 3 (CD44^hi^NK1.1^+^). Data are representative of three independent analyses with total six mice per group. **(C)** Flow-cytometric analysis of expression of PLZF and T-bet on gated thymic iNKT cells from control and IL-4KO mice. Numbers adjacent to outlined areas and the graph show percent iNKT cells divided into subsets A (PLZF^+^) and B (T-bet^+^). Data are representative of three independent analyses with total six mice per group. **(D)** Flow-cytometric analysis of expression of CD4 and CD8 on gated thymic iNKT cells from control and IL-4KO mice. Numbers in quadrants and graphs show percent of CD4^+^CD8^−^ and CD4^−^CD8^−^ iNKT cells. Data are representative of three independent analyses with total six mice per group.

In analogy with Th2 cells that produce IL-4 and utilize the cytokine for maintenance of differentiated status, we questioned if IL-4 plays an autocrine role in promoting the generation and/or maintenance of PLZF-expressing iNKT cells. To this end we assayed for the CD1d-restricted thymocytes in IL-4-deficient mice. Staining of total thymocytes for CD1d tetramers loaded with glycolipid α-galactosylceramide analogue PBS-57 (CD1d-PBS-57) and TCRβ showed that thymic iNKT cell frequency and absolute numbers were comparable in wild-type control and IL-4KO mice **(** **Fig. 1A** **)**. These data demonstrate that IL-4 deficiency does not impair iNKT cell development in the thymus.

iNKT cells develop through stages 1 (CD44^lo^NK1.1^−^), 2 (CD44^hi^NK1.1^−^) and 3 (CD44^hi^NK1.1^+^) [2], [21]. To determine if IL-4 regulates the developmental stages of iNKT cells, we stained thymocytes from control and IL-4KO thymocytes with CD1d-PBS-57, anti-TCRβ, anti-CD44 and anti-NK1.1 antibodies. Analysis of tetramer positive thymocytes showed the frequency of maturing IL-4KO iNKT cells was similar to control thymic iNKT cells and that the majority of the thymic iNKT cells were found to be at stage 3 of development **(** **Fig. 1B** **)**. The frequency of thymic iNKT cells at stage 1, 2 and 3 was very similar in control and IL-4KO mice **(** **Fig. 1B** **)**. These data show that IL-4 expression is not required for differentiation of iNKT cell stages or for survival of developing cells at any stage.

As CD1d-restricted TCRβ^+^ iNKT cells can be subdivided into A and B subsets based on the expression of transcription factors PLZF or T-bet, we characterized the IL-4-deficient thymic iNKT cell subset distribution by intracellular staining with antibodies to PLZF and T-bet. Expression of these transcription factors was comparable in iNKT cells from control and IL-4KO mice **(** **Fig. 1C** **)**, indicating that functional subsets defined by critical transcription factor expression are not affected by absence of IL-4 expression.

Finally, the iNKT cell population consists of both CD4^+^CD8^–^ (CD4^+^) and CD4^–^CD8^–^ double negative (DN) cells [2], [22]. We note that in IL-4-deficient thymus, the frequency and numbers of iNKT cell distribution in both CD4^+^ and DN subsets were similar to those in control thymus **(** **Fig. 1D** **)**. Together these studies demonstrate that IL-4 deficiency does not affect iNKT cell development or survival in the thymus.

### IL-4 deficiency does not affect iNKT cell migration to or maintenance in peripheral organs

**Figure 2:**
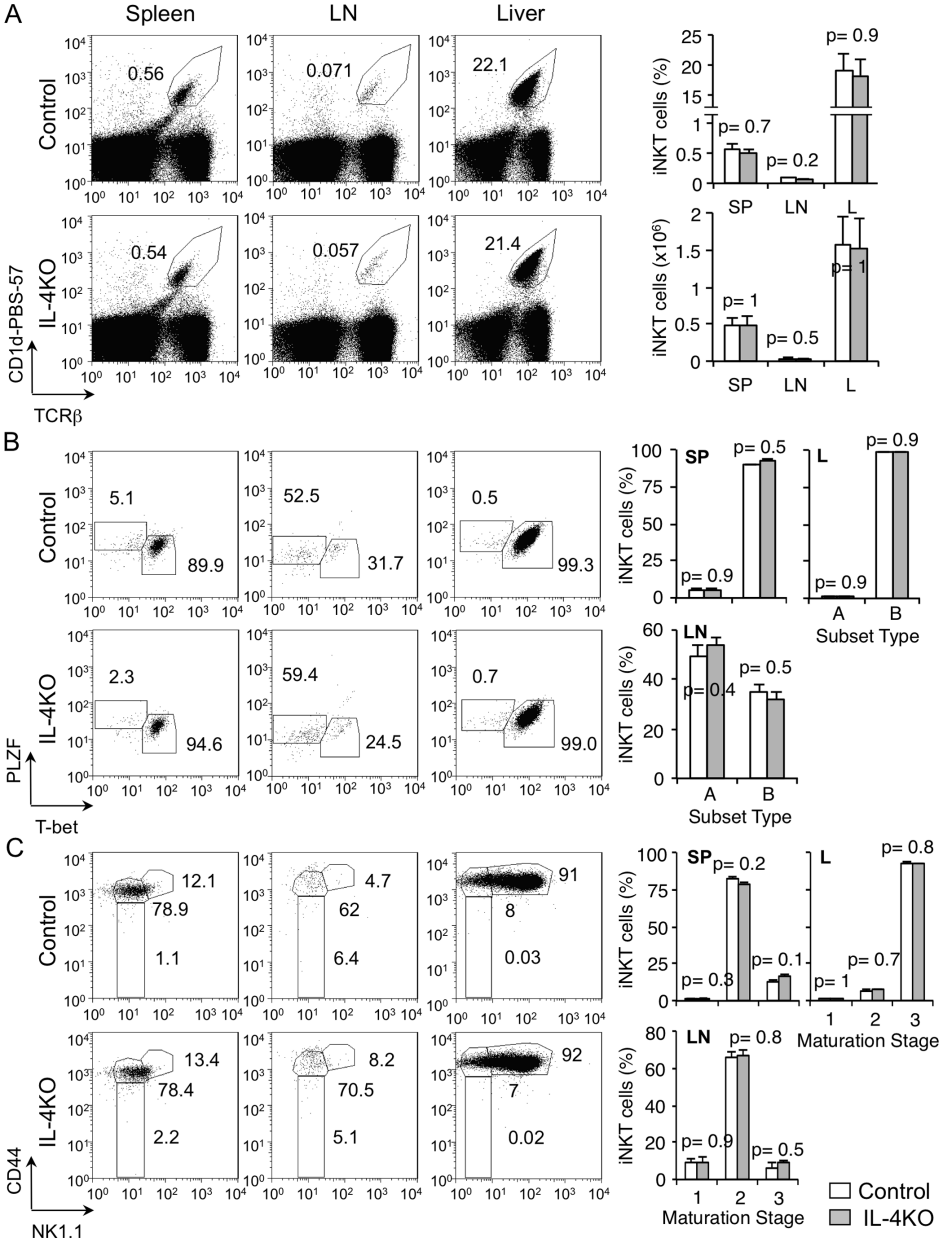
Peripheral iNKT cells are not affected by IL-4 deficiency. (**A**) Flow-cytometric analysis of expression of CD1d-PBS-57 and TCRβ on total lymphocytes from spleen, lymph nodes (LNs) and liver of control and IL-4KO mice. Numbers adjacent to outlined areas and the graphs show percent of total iNKT cells for each organ as indicated. Data are representative of three independent analyses with total 4–6 mice per group. **(B)** Flow-cytometric analysis of expression of PLZF and T-bet on gated iNKT cells from spleen, LNs and liver of control and IL-4KO mice. Numbers adjacent to outlined areas and the graphs show percent iNKT cells divided into subsets A (PLZF^+^) and B (T-bet^+^). Data are representative of three independent analyses with total 4–6 mice per group. **(C)** Flow-cytometric analysis of expression of CD44 and NK1.1 on gated iNKT cells from spleen, LNs and liver of control and IL-4KO mice. Numbers adjacent to outlined areas and the graphs show percent of gated iNKT cells divided into maturation stages 1, 2 and 3. Data are representative of three independent analyses with total 4–6 mice per group.

To determine if IL-4 deficiency affects peripheral distribution of iNKT cells, we stained for the CD1d-PBS-57 binding T cells in the spleen, lymph nodes (LNs) and liver of IL-4-deficient mice. The frequency of iNKT cells in the spleen, LNs and liver of IL-4KO mice was similar to that in control mice **(** **Fig. 2A** **)**. To further characterize the iNKT cells that accumulate in spleen, LNs and liver, we identified CD1d-restricted T lymphocytes and assessed PLZF or T-bet expression. Similar to control mice, the majority of T-bet-expressing subset B iNKT cells were found in the spleen and liver and PLZF-expressing subset A iNKT cells were found in the LNs of IL-4KO mice **(** **Fig. 2B** **)**. Next we stained peripheral lymphocytes for stages of iNKT development; we found that CD1d-restricted CD44^hi^ NK1.1^−^ iNKT cells at stage 2 of development accumulated in the spleen and LNs and CD44^hi^ NK1.1^+^ stage 3 iNKT cells accumulated in the liver of control and IL-4KO mice **(** **Fig. 2C** **)**. These data demonstrate that IL-4 deficiency does not influence the distribution of iNKT cells to the peripheral lymphoid tissues or further development of these cells in the peripheral tissues. We therefore conclude that IL-4 is not required for the distribution of iNKT cells.

### IL-4Rα expression is dispensable for the development of iNKT cells in the thymus

**Figure 3:**
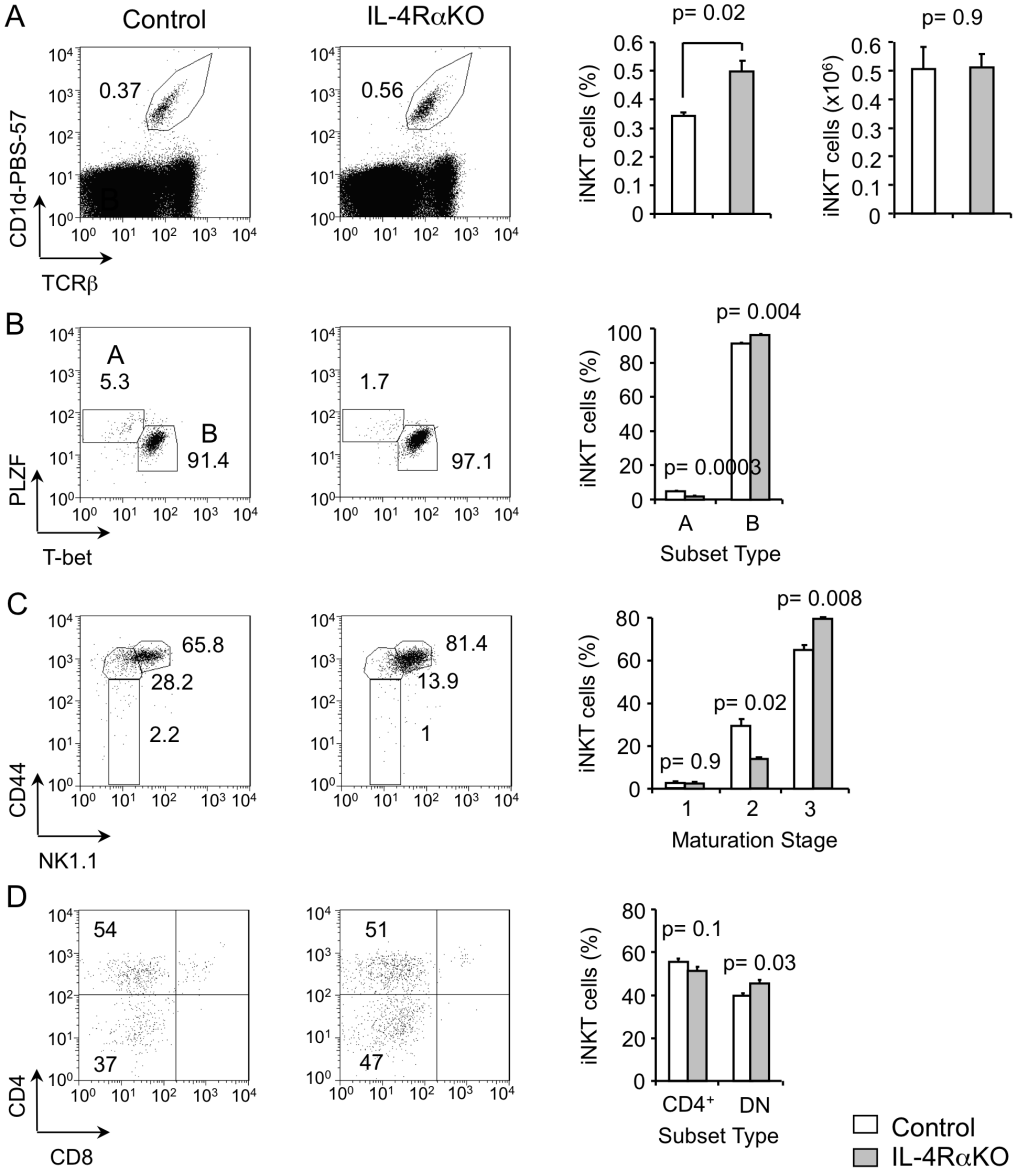
IL-4Rα expression is not required for the development of thymic iNKT cells. (**A**) Flow-cytometric analysis of expression of CD1d-PBS-57 and TCRβ on total thymocytes from control and IL-4RαKO mice. Numbers adjacent to outlined areas and the graphs show percent and cell numbers of total iNKT cells as indicated. Data are representative of three independent analyses with total six mice per group. **(B)** Flow-cytometric analysis of expression of PLZF and T-bet on gated thymic iNKT cells from control and IL-4RαKO mice. Numbers adjacent to outlined areas and the graph show percent iNKT cells divided into subsets A (PLZF^+^) and B (T-bet^+^). Data are representative of three independent analyses with total six mice per group. **(C)** Flow-cytometric analysis of expression of CD44 and NK1.1 on gated thymic iNKT cells from control and IL-4RαKO mice. Numbers adjacent to outlined areas and graphs show percent of gated iNKT cells divided into maturation stages 1, 2 and 3. Data are representative of three independent analyses with total six mice per group. **(D)** Flow-cytometric analysis of expression of CD4 and CD8 on gated thymic iNKT cells from control and IL-4RαKO mice. Numbers in quadrants and graphs show percent of CD4^+^CD8^−^ and CD4^−^CD8^−^ iNKT cells. Data are representative of three independent analyses with total six mice per group.

Next, we wanted to determine if other cytokines might substitute for IL-4 in its absence in iNKT cell development or distribution. To accomplish this, we analyzed iNKT cells in IL-4Rα-deficient mice. We found that there is a slight increase in the frequency of iNKT cells in IL-4Rα-deficient thymus compared to control thymus **(** **Fig. 3A** **)**. However, the absolute numbers were comparable to control **(** **Fig. 3A** **)**. Further characterization showed that IL-4Rα deficiency resulted in a slight but significant increase in the frequency and numbers of T-bet-expressing iNKT cells **(** **Fig. 3B** **)** that were at developmental stage 3 **(** **Fig. 3C** **)**. We note a corresponding modest but significant decrease in the PLZF-expressing stage 2 iNKT cells **(** **Fig. 3B and 3C** **)**. These data suggest that IL-4Rα signals might regulate the balance between PLZF-expressing and T-bet-expressing iNKT cells in the thymus, shifting it slightly towards T-bet. Mechanisms involved in generating this balance will be an interesting topic for future studies.

We also found a slight increase in the frequency of DN iNKT cells and a decrease in CD4^+^ iNKT cells in IL-4Rα-deficient thymus compared to control subset distribution, but the numbers were not statistically significant **(** **Fig. 3D** **)**. Together these data demonstrate that IL-4Rα-dependent signals are generally dispensable for the development of iNKT cells in the thymus.

### IL-4R signaling is dispensable for the maintenance of iNKT cells in peripheral organs

**Figure 4:**
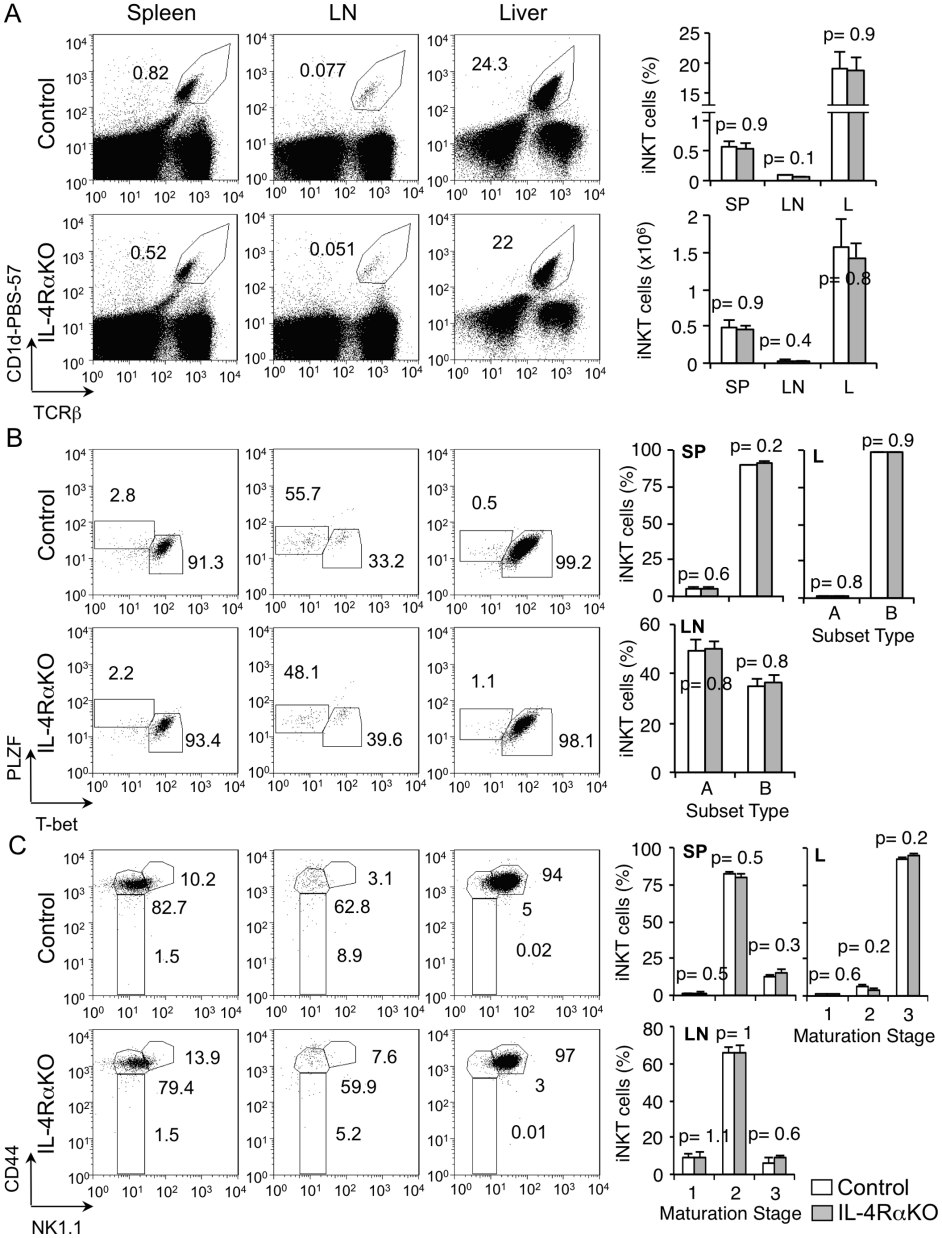
Peripheral iNKT cells are not affected by IL-4Rα deficiency. (**A**) Flow-cytometric analysis of expression of CD1d-PBS-57 and TCRβ on total lymphocytes from spleen, LNs and liver of control and IL-4RαKO mice. Numbers adjacent to outlined areas and the graphs show percent of total iNKT cells for each organ as indicated. Data are representative of three independent analyses with total 4–6 mice per group. **(B)** Flow-cytometric analysis of expression of PLZF and T-bet on gated iNKT cells from spleen, LNs and liver of control and IL-4RαKO mice. Numbers adjacent to outlined areas and the graphs show percent iNKT cells divided into subsets A (PLZF^+^) and B (T-bet^+^). Data are representative of three independent analyses with total 4–6 mice per group. **(C)** Flow-cytometric analysis of expression of CD44 and NK1.1 on gated iNKT cells from spleen, LNs and liver of control and IL-4RαKO mice. Numbers adjacent to outlined areas and the graphs show percent of gated iNKT cells divided into maturation stages 1, 2 and 3. Data are representative of three independent analyses with total 4–6 mice per group.

The frequency of iNKT cells in the spleen, LNs and liver of IL-4RαKO mice was similar to that in control mice **(** **Fig. 4A** **)**. Further characterization showed that spleen and liver iNKT cells were T-bet-expressing subset B in majority and LN iNKT cells were PLZF-expressing subset A with comparable frequencies in IL-4RαKO mice and control mice **(** **Fig. 4B** **)**. Similar to control, CD44^hi^ NK1.1^−^ iNKT stage 2 cells were found in the spleen and LNs and CD44^hi^ NK1.1^+^ stage 3 iNKT cells were found in the liver of IL-4RαKO mice **(** **Fig. 4C** **)**. We conclude that IL-4Rα is not critical for the maintenance of iNKT cells or their further maturation in peripheral lymphoid organs.

### IL-4- or IL-4Rα-deficiency do not impair iNKT cell activation upon stimulation

**Figure 5:**
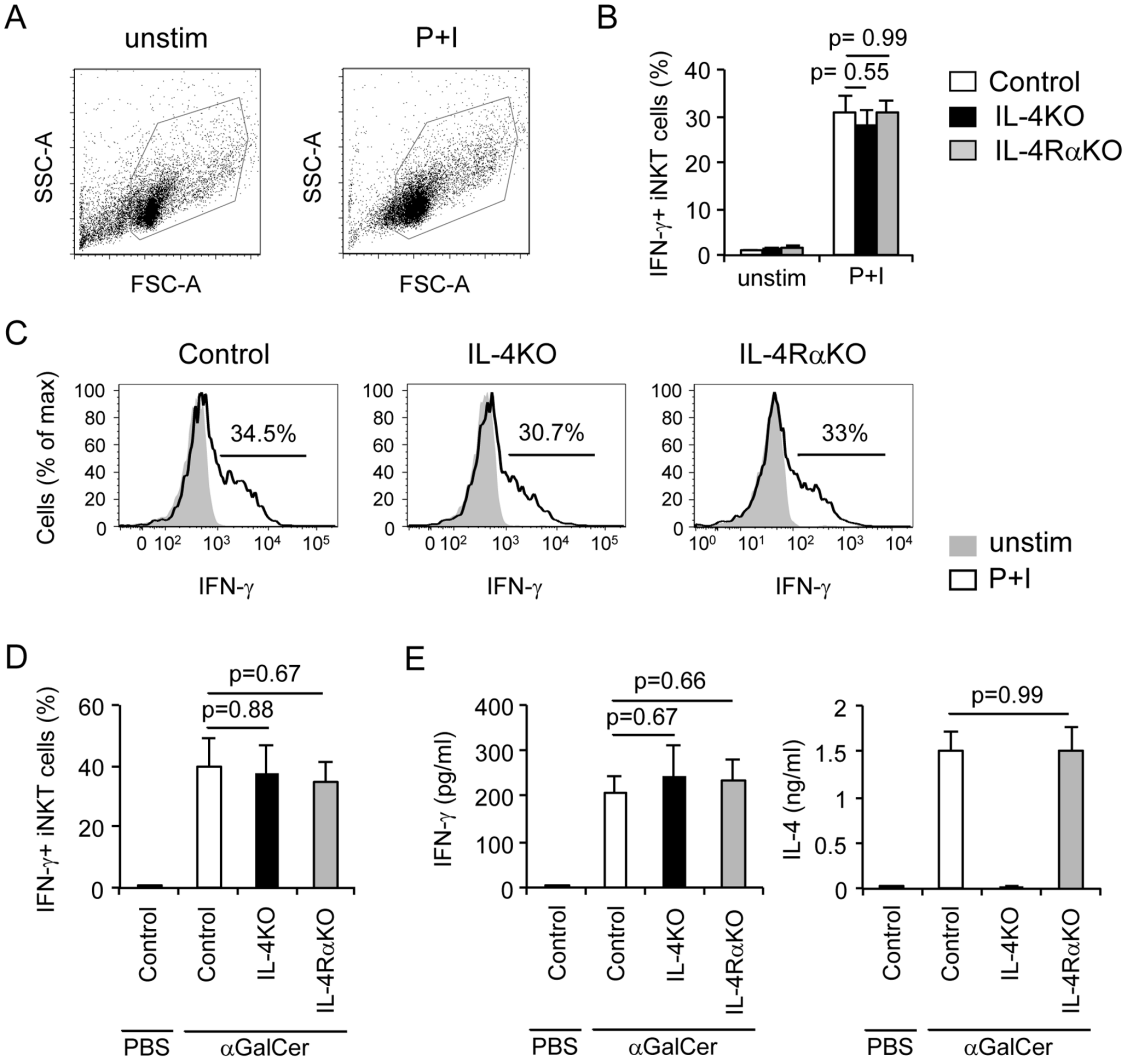
Stimulated iNKT cells produce IFN-γregardless of IL4 or IL-4Rα deficiency. (**A–C**) Total thymocytes were left unstimulated or stimulated with PMA (50 ng/ml) and ionomycin (1 µM) (P+I) for 5 hours from control, IL-4KO and IL-4RαKO mice. Brefeldin A was added for the last 3.5 hours. Data are representative of total six mice per group. **(A)** Flow-cytometric analysis of size (FSC-A) and complexity (SSC-A) of total thymocytes unstimulated or stimulated with P+I from one representative mouse. **(B)** Graphic representation of percent of IFN-γ positive thymic iNKT cells from control, IL-4KO and IL-4RαKO mice, as indicated. Data are representative of six mice per group. **(C)** IFN-γ intracellular expression by thymic iNKT cells from unstimulated (shaded) or stimulated with P+I (open) of control, IL-4KO and IL-4RαKO mice. Numbers in plots indicate percent of IFN-γ positive cells. **D–E)** Control, IL-4KO and IL-4RαKO mice were analized 3 hours after i.p. injection of 3 µg αGalCer or PBS. Data are representative of total six mice per group. **(D)** Graphic representation of percent of IFN-γ positive splenic iNKT cells. **(E)** IFN-γ and IL-4 production detected by ELISA using serum of stimulated mice.

To determine if IL-4 or IL-4R signaling is required for the development of effector functions in iNKT cells, we assayed the cytokine production by iNKT cells from control, IL-4KO and IL-4RαKO mice. To assess whether iNKT cells rapidly produce cytokines in the absence of either IL-4 or IL-4Rα, we stimulated *in vitro* IL-4KO, IL-4RαKO and control thymocytes for 5 hours with PMA and ionomycin and used intracellular staining to determine the percentage of iNKT cells that produced IFN-γ. We note that reports in the literature show that cytokine production by iNKT cells is variable [23], [24]. We found that IFN-γ production by control and IL-4KO and IL-4RαKO iNKT cells was comparable and our values were within the range described in the literature (**Fig. 5A–C**). These data show that IL-4 or IL-4Rα expression is not required for rapid cytokine production by iNKT cells.

Next we assessed cytokine production by IL-4- and IL-4Rα-deficient iNKT cells after stimulation *in vivo*. For this experiment we administered the iNKT cell antigen αGalCer (3 µg, i.p.) to IL-4KO, IL-4RαKO and control mice and assayed cytokine production 3 hours later. We found comparable frequencies of IFN-γ positive iNKT cells from spleen (**Fig. 5D**) and liver (data not shown) of IL-4KO, IL-4RαKO and control mice. Even though we note minor quantitative differences between mice from the same genotype, we found no significant differences among the different types of mice analyzed. Finally, we quantified the levels of IFN-γ and IL-4 in serum of the mice (**Fig. 5E**). Once again, we encountered some mouse-to-mouse variability within each strain but no significant differences between IL-4KO, IL-4RαKO and control mice. Therefore, these data clearly demonstrate that neither IL-4 nor IL-4Rα expression is required for the antigen-dependent activation of iNKT cells to rapidly produce cytokines *in vitro* and *in vivo*.

## Discussion

In this paper we report that expression of IL-4 and IL-4Rα is dispensable for the development, maintenance and functional maturation of iNKT cells. We demonstrate that the frequency and absolute numbers of iNKT cells in the thymus in IL-4- and IL-4Rα-deficient mice are comparable to wild-type control mice. Similar to control, iNKT cells develop normally through stages 1, 2 and 3 in IL-4- and IL-4Rα-deficient mice. Our data demonstrate that the number of iNKT cells in peripheral lymphoid organs spleen, LNs and liver in IL-4- and IL-4Rα-deficient mice was also comparable to control mice. We also show that IL-4 deficiency does not exert any effect on the expression patterns of the transcription factors PLZF and T-bet, which are involved in the development and differentiation of thymic iNKT cells. However, we do note that the frequency of PLZF expressing iNKT cells is slightly decreased and the frequency of T-bet expressing cells is increased when iNKT cells lack IL-4Rα chain expression. Finally, we demonstrate that neither IL-4 nor IL-4Rα signaling are required for the effector function of iNKT cells as antigen-stimulated IL-4KO, IL-4RαKO and control iNKT cells rapidly produce comparable cytokine amounts *in vitro* and *in vivo*. Thus, we show that iNKT cell development and functional maturation is independent of IL-4 and IL-4Rα signaling.

CD4^+^ iNKT cells produce high levels of IL-4 and both CD4^+^ and DN iNKT cells produce high levels of IFN-γ [22], [25], [26]. As conventional Th1 cells utilize IFN-γ and Th2 cells utilize IL-4 in an autocrine manner to induce and maintain differentiated status [16], [17], we wondered if IL-4 produced by CD4^+^ iNKT cells is required for the development and maintenance of these cells. Data presented in this paper shows that IL-4 and IL-4Rα signaling is dispensable for the development, differentiation and maintenance of iNKT cells. Signaling requirements for IL-4 production by Th2 cells and iNKT cells have been found to be distinct. Specifically, IL-4 expression is independent of IL-4Rα/STAT6 signaling in iNKT cells and GATA-3, which is normally induced by STAT6 activation, is expressed at low levels in iNKT cells [27]. IL-4Rα/STAT6 signaling-dependent IL-13 produced by iNKT cells plays an important role in iNKT cell-dependent tumor immunosurveillance [28]. Whereas published data show the regulation of IL-4 production by iNKT cells, they do not address the issue of a requirement for IL-4Rα signaling in iNKT cell development, maintenance and homeostasis. Our observation that the frequency of PLZF- or T-bet-expressing iNKT cells is influenced by the absence of IL-4Rα chain expression suggests that IL-4/IL-4Rα chain-dependent signals might influence iNKT cell function under certain conditions. Mechanisms involved in generating this balance will be an interesting topic for future studies. By comparing IL-4- and IL-4Rα-deficient mice with control mice, we demonstrate that IL-4R signaling is expendable for iNKT cell development and function. Thus, we conclude that the data presented in this report demonstrate that both IL-4 and IL-4Rα signaling pathways are dispensable for the development, maintenance and functional differentiation of iNKT cells.

## Materials and Methods

### Animals

IL-4-deficient mice were obtained from The Jackson Laboratory and IL-4Rα-deficient mice [29] were provided by Z. Zhu (The Johns Hopkins Asthma and Allergy Center, MD). All mice were on C57BL/6 genetic background. Mice of 7–9 weeks old were used for analysis. Age-matched littermate controls or C57BL/6 mice were used in all experiments. All mice were bred and maintained in animal facility at the National Institute on Aging (NIA). The studies were carried out in accordance with the recommendations in the Guide for the Care and Use of Laboratory Animals (NRC 2010). The protocol was approved by the Animal Care and Use Committee of the NIA Intramural Research Program, NIH. This program is fully accredited by the Association for Assessment and Accreditation of Laboratory Animal Care International (AAALAC) (File 000401), registered by the United States Department of Agriculture (51-F-0016) and maintains an assurance with the Public Health Service (A4149-01).

### Cell Preparation

Single-cell suspensions were prepared from thymus, lymph nodes and spleens as per standard protocols. Hepatic lymphocytes were isolated from livers that were homogenized and washed in PBS with 1% FBS and filtered through nylon mesh. Cells were then re-suspended in 44% Percoll (GE Healthcare Bio-Sciences AB, Uppsala, Sweden), under-laid with 66% Percoll, and centrifuged for 20 min at 2000 rpm. Cells at the interface were collected, washed, and counted.

### Antibodies and flow cytometry

Cells were harvested, stained and analyzed on a FACS Calibur or Canto II (Becton Dickinson). Dead cells were excluded by forward light scatter or forward light scatter plus propidium iodide. All the data were acquired and are presented on log scale. The following antibodies conjugated to FITC, PE, peridinin chlorophyll protein-cyanine 5.5, or allophycocyanin, all purchased from BD Biosciences or eBioscience, were used for staining: anti-CD4 (GK1.5), anti-CD8α (53–6.7), anti-CD44 (IM7), anti-TCRβ (H57-597), anti-NK1.1 (PK136) and anti-IFN-γ (XMG1.2). PE- or eFluor660- conjugated anti-T-bet (eBio4B10) antibodies were purchased from eBioscience. PE- or allophycocyanin- conjugated mouse CD1d tetramers loaded with glycolipid PBS-57 (CD1d-PBS-57) were obtained from the tetramer facility of the US National Institutes of Health. PLZF staining was done as described [5]. In brief, cell surfaces were stained with antibodies, and then cells were fixed with the Foxp3 Staining Buffer set (eBioscience). Permeable cells were incubated with antibody to PLZF (D-9; Santa Cruz) followed by anti-mouse immunoglobulin G1 (A85-1; BD Biosciences) alone or with anti-T-bet (eBio4B10) where appropriate.

### In vitro PMA- and ionomycin-induced activation assay

For *in vitro* stimulation, thymocytes were cultured in T cell medium (RPMI 1640 with 10% FBS, HEPES, penicillin and streptomycin, _L_-glutamine and 2-mercaptoethanol) and stimulated for 5 hours with phorbol 12-myristate 13-acetate (50 ng/ml) and ionomycin (1 µM). For intracellular cytokine staining, Brefeldin A was added for the final 3.5 hours. Cells were stained with anti-TCRβ and CD1d-PBS-57, and with anti-IFN-γ (Cytofix/Cytoperm kit from BD Biosciences) following the manufacturer’s instructions.

### In vivo αGalCer stimulation

αGalCer 3 µg in 200 µl PBS were intraperitoneally injected into mice. Mice were bled and sacrificed to obtain spleen 3 hours after injection. Blood was allowed to clot and the serum was prepared from the clots. Quantity of IFN-γ and IL-4 was measured using ELISA (ELISA Ready-SET-Go; eBioscience) following the manufacturer’s instructions. For the intracellular IFN-γ expression, splenocytes were stained using the Cytofix/Cytoperm kit from BD Biosciences.

### Statistics

Statistical significance was determined by the Student t-test.
